# Beyond tumour suppression: cGAS‐STING pathway in urologic malignancies: Context‐dependent duality and therapeutic implications

**DOI:** 10.1002/ctm2.70531

**Published:** 2025-11-23

**Authors:** Qi Wei, Kui Zhao, Yifan Wu, Wenhui Wu, Na Hao

**Affiliations:** ^1^ Department of Oncology First Teaching Hospital of Tianjin University of Traditional Chinese Medicine Tianjin China; ^2^ National Clinical Research Center for Chinese Medicine Acupuncture and Moxibustion Tianjin China; ^3^ National Research Institute for Family Planning Beijing China; ^4^ Cell Biology Chinese Academy of Medical Sciences & Peking Union Medical College Beijing China; ^5^ Department of Nephrology Second Affiliated Hospital of Tianjin University of Traditional Chinese Medicine Tianjin China

**Keywords:** cGAS–STING, dual regulatory role, mechanisms, therapies, urologic cancers

## Abstract

**Background:**

The cyclic GMP–AMP synthase (cGAS)–stimulator of interferon (IFN) genes (STING) pathway emerges as a dual‐functional role in urologic malignancies, exhibiting context‐dependent tumor‐suppressive and pro‐tumorigenic activities. When this pathway is activated in urologic tumors, IFN transcription and CD8^+^ T cell infiltration are triggered, which has an anticancer effect. However, this pathway facilitates the development of prostate cancer through the up‐regulation of regulatory B cells. STING palmitoylation triggers immune escape in renal cell carcinoma, and the STING/SLC14A1 axis also mediates chemoresistance in bladder cancer.

**Main topics covered:**

Based on these findings, we establish the first systematic comparison of tissue‐specific STING regulation in urological malignancies, challenging the conventional tumor suppressor‐centric view. This review also highlights several innovative strategies leveraging this duality during urologic cancers. The demand for the long‐term safety and effectiveness of these targeted STING treatments has not been fully met.

**Conclusions:**

This study introduces a framework that harnesses the dual functions of the cGAS‐STING pathway to strengthen immunotherapy approaches and improve clinical outcomes to bridge the pre‐clinical‐clinical gap.

**Key points:**

- The context‐dependent duality of cGAS–STING signalling in urologic tumours is revealed.
- Targeting the STING pathway, in combination with immunotherapies and gene therapies, enhances the anti‐tumour response.
- Sex hormone differences in urological malignancies are correlated with the cGAS–STING pathway.

## Graphical abstract

1. The context‐dependent duality of cGAS–STING signalling in urologic tumours is revealed.
2. Targeting the STING pathway, in combination with immunotherapies and gene therapies, enhances the anti‐tumour response.
3. Sex hormone differences in urological malignancies are correlated with the cGAS–STING pathway.

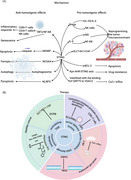

## 1 INTRODUCTION

Urologic malignancies, including prostate cancer (PCa), renal cell carcinoma (RCC) and bladder cancer (BCa), constitute a growing global burden of disease. GLOBOCAN 2022 data reveal more than 2.5 million annual new diagnoses and 770 000 annual deaths.^1^ PCa ranks second in the number of diagnosed cases among men and accounts for the fifth among cause of cancer fatalities in males, whereas RCC is the 10th most frequent type of tumour among women.^1^ It is also important to note that BCa exhibits a striking sex‐specific disparity: a 3.9:1 male‐to‐female incidence ratio.^1^ These epidemiological patterns highlight the pressing requirement for improved treatments. The current standard of care, such as radiochemotherapy and immunotherapy, faces critical limitations due to tumour immune escape mechanisms. This phenomenon is attributed to the dysfunction of the immune circuit characterised by an increase in pro‐tumourigenic M2‐like macrophages as well as exhausted CD8^+^ T cells.^2^ Compounding this challenge, many urologic tumours exhibit a ‘cold’ immunophenotype that evades immune surveillance by secreting TGF‐β, IL‐6 and IL‐35.^3^ Overall, tumour immune escape is a key impediment to efficacious immunotherapy, necessitating innovative therapeutic paradigms.

Emerging evidence positions the cyclic GMP–AMP synthase (cGAS)–stimulator of interferon (IFN) genes (STING) pathway as a viable target in tumour immunotherapy, given its pivotal function in facilitating T lymphocytes.^4^, ^5^ This pathway orchestrates the innate immune system through IFN induction.^6^ In the innate immune system, STING moves from the endoplasmic reticulum (ER) to the Golgi, where it oligomerises and enlists TANK‐binding kinase 1 (TBK1), ultimately contributing to activation of IFN regulatory factor 3 (IRF3) and nuclear factor κB (NF‐κB). This signalling cascade culminates in the production of type I IFNs (including IFN‐α and IFN‐β), type III IFNs (IFN‐λ) and pro‐inflammatory cytokines, activating the function of T lymphocytes.^4^, ^7^, ^8^ In addition, IFN‐α generated by the STING signalling pathway mediates the expression of type II IFN (IFN‐γ) by inducing the activation of natural killer (NK) cells, thereby promoting the anti‐tumour effect.^9^ Most importantly, the interaction between therapeutic approaches targeting this pathway and urologic tumours has been identified. STING agonist/ inhibitors and a vector carrying the STING activator reverse immunosuppression in these tumours through facilitating the expression of inflammatory cytokines, indicating their therapeutic potential.^10^, ^11^, ^12^

This review synthesises emerging insights into bidirectional regulation of the cGAS–STING pathway in urologic malignancies. The context‐dependent effects of STING activation or inhibition across different tumour types have been elucidated. Emphasis is placed on disease‐specific pathogenesis of each urologic malignancy. Notably, STING‐targeted therapies offer novel avenues to overcome treatment resistance. STING agonists/inhibitors boost immune response to potentiate the efficacy of chemoradiotherapy and immunotherapy. Vectors carrying the STING activator, such as lipid nanoparticles (LNPs) and irradiated tumour cell‐derived microparticles (RMPs), deliver STING agonists to specific sites of urologic malignancies to enhance cytokine release, thus maximising therapeutic efficacy and minimising systemic toxicity. This reciprocal regulation of the cGAS–STING pathway challenges the traditional cancer suppressor‐centric perspectives. By clarifying the mechanistic link between this pathway and tumour–immune crosstalk, this study provides a translational framework for developing precision interventions against tumour immune escape in urologic cancers as well as extending the clinical benefits through combination therapy.

## 2 HISTORY OF cGAS–STING PATHWAY

cGAS, a nucleotidyltransferase, comprises an N‐terminal domain along with a compact catalytic domain.^13^ Residing predominantly in the cytosol, cGAS exists as a dimer that maintains a stable conformation by clamping double‐stranded DNA (dsDNA).^14^ It recognises dsDNA from diverse sources, including bacteria, viruses, protozoan parasites and mitochondria, and mediates the assembly of cGAS polymer via liquid–liquid phase separation, thereby promoting its activation.^15^, ^16^ Although primarily cytoplasmic, cGAS has been detected at the plasma membrane and inside the nucleus.^17^ At the plasma membrane, the N‐terminal domain of cGAS binds phosphatidylinositol 4,5‐bisphosphate (PIP2) in the inner leaflet, facilitating more efficient detection of viral DNA while suppressing aberrant responses to endogenous DNA.^18^ However, an emerging study indicates that endogenous cGAS is predominantly enriched within the nucleus.^17^ Immunofluorescence microscopy reveals significantly higher nuclear signal intensity compared with the cytosol.^17^ This altered localisation may be linked to nuclear envelope breakdown (NEBD) during mitosis.^19^ Specifically, prior to NEBD, cGAS accumulates at the nuclear periphery; following rupture, it associates with centromeric DNA via its N‐terminal residues 1–160, leading to its accumulation on chromatin. Nuclear cyclic guanosine monophosphate‐adenosine monophosphate (cGAMP), synthesised by chromatin‐bound cGAS, diffuses or is actively transported through nuclear pores into the cytosol, where it activates STING and downstream signalling pathways.^19^

STING is recognised as an ancient adapter transmembrane protein in ER.^20^ It possesses an N‐terminal cytoplasmic segment, four transmembrane helical regions, a cytoplasmic ligand‐binding domain (LBD) and a disordered C‐terminal tail (CTT).^21^ Its stability is dependent on the interaction with the ER‐resident protein stromal interaction molecule 1 (STIM1), which maintains STING in a quiescent state.^22^ In its basal state, STING forms stable dimers within the ER via its LBD.^23^ The interaction of cGAMP triggers a structural alteration in the STING–LBD, promoting exposure of the CTT and facilitating its interaction with TBK1.^24^ The activation of TBK1 results in the phosphorylation of STING–CTT, creating a docking site for IRF3 and driving IRF3 dimerisation.^25^ Importantly, the functional activity of STING is critically dependent on its palmitoylation.^26^, ^27^ This protein modification occurs primarily on cysteine (Cys) residues.^28^ Specifically, mutation of the Cys88 and Cys91 sites in STING markedly reduces palmitoylation levels and strongly suppresses IFN‐I response.^26^ Consistently, the palmitoylation inhibitor 2‐bromohexadecanoic acid (2‐BP) significantly abolishes IFN‐I expression by specifically targeting STING palmitoylation.^26^ Overall, these findings indicate that palmitoylation plays an indispensable role in regulating STING‐mediated innate immune signalling.

Fundamental to the early investigation was that STING mounts robust IFN‐β in response to numerous pathogens.^29^ Subsequent investigations in 2013 revealed that this response is triggered by direct binding of STING to cGAMP.^30^ Notably, some DNA sensors have the capability to trigger STING pathway activation, further amplifying IFN signalling.^31^ Until the discovery of cGAS, the upstream regulator of STING pathway has been identified.^32^ It has been demonstrated that cGAS recognises DNAs and subsequently recruits cGAMP, initiating downstream signalling of STING pathway.^32^

## 3 ACTIVATION OF THE cGAS–STING PATHWAY

**FIGURE 1:**
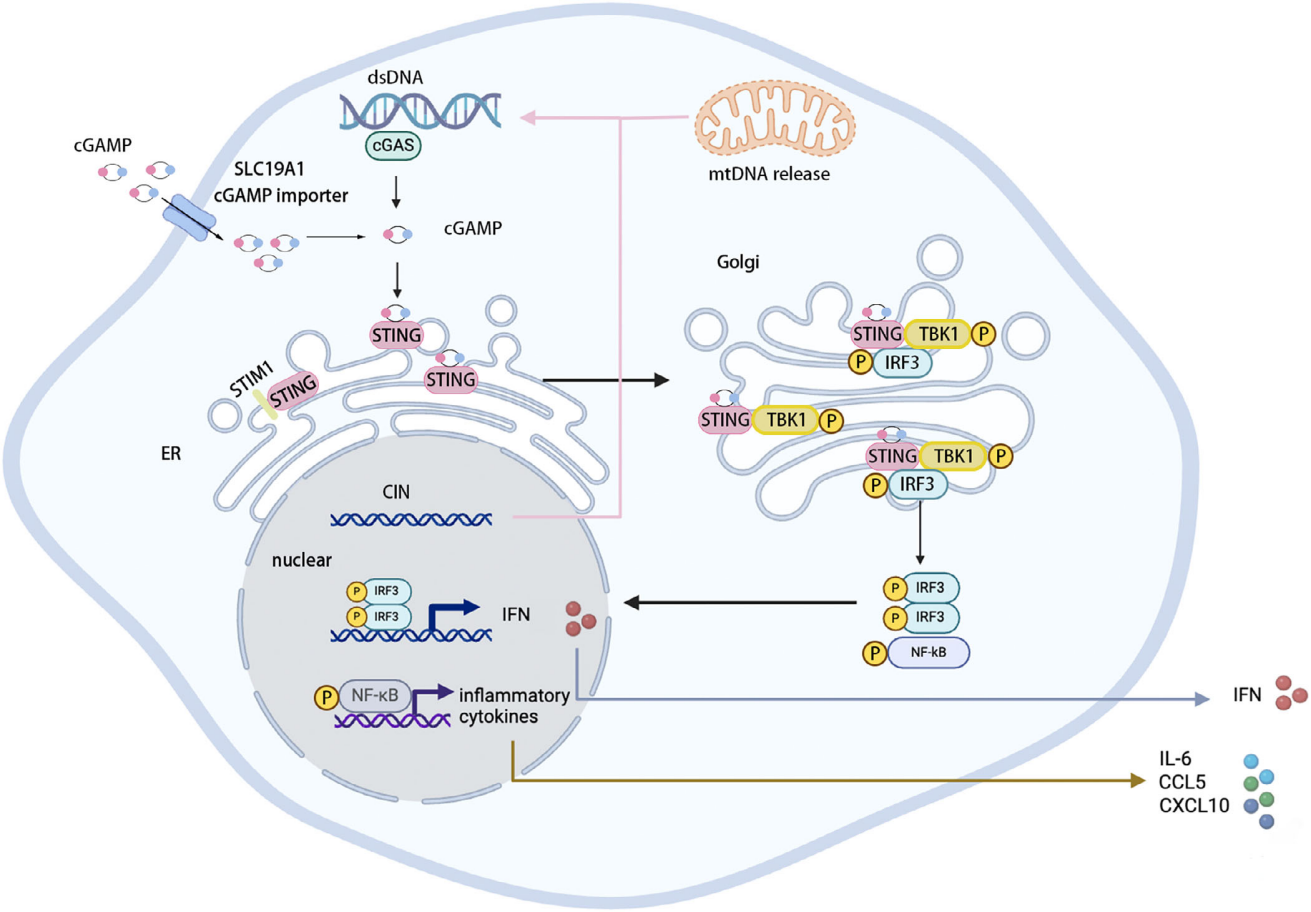
Overview of cGAS–STING pathway. Upon stimulation by physical or chemical factors, an imbalance between DNA damage and repair mechanisms occurs. Cytoplasmic release of damaged DNA fragments can be detected by cGAS. This recognition induces conformational changes in cGAS, forming a catalytic pocket that facilitates the synthesis of cGAMP. Binding of cGAMP to the STING–LBD terminates the dormant ER‐associated conformation of STING (dependent on STIM1 binding) and triggers its relocation to the Golgi. Within the Golgi, STING initiates TBK1 activation and recruitment, forming a STING–TBK1 complex that subsequently associates with IRF3. This ternary complex facilitates nuclear translocation of IRF3 and NF‐κB, which ultimately induces the production of IFN and various chemokines.

Mitochondrial DNA (mtDNA), cytoplasmic dsDNA, nuclear DNA or extracellular DNA is released into the cytoplasm. Once there, cGAS recognises and binds to it (Figure 1). This recognition triggers the synthesis of cGAMP.^33^ Central to this signalling is STING, an ER‐resident transmembrane adapter protein.^34^ After cGAMP binds to STING, the STING–STIM1 complex is disrupted, inducing conformational changes and oligomerisation of STING. The STING oligomers promote phosphatidylinositol 3‐phosphate accumulation, which mediates the curvature of ER membrane and the formation of COPII vesicles. This process facilitates STING transport from the ER, through the ER–Golgi intermediate compartment (ERGIC), to the Golgi.^35^ At the Golgi membrane, STING recruits and is phosphorylated by the robust TBK1.^36^ This interaction allows IRF3 to bind STING, forming a STING–TBK1–IRF3 complex.^37^ Concurrently, the STING oligomers recruit the IKK complex, triggering NF‐κB activation.^25^ It should be emphasised that the activation mechanism of NF‐κB is intricate and may involve not only the STING–CTT but also its LBD.^13^ Subsequently, IRF3 and NF‐κB move to the nucleus, driving transcription of IFN and diverse cytokines, respectively.^38^, ^39^ IFN establishes an immunostimulatory microenvironment through enhancing the activation of T cells and NK cells.^40^ These cytokines, including CCL5 and CXCL10, up‐regulate tumour cell lysis via large amounts of granzyme B.^40^, ^41^ Collectively, the activation of the classical pathway results in immunosuppression against tumours.

The cGAS–STING pathway mediates tumour immune surveillance by modulating immune responses. However, its role in tumour progression exhibits remarkable context‐dependent duality. This dual nature is governed not only by the mode of pathway activation (canonical vs. noncanonical) but also by the intrinsic characteristics of tumours. The following section will systematically dive deep into the characteristics of tumours, thereby providing essential context for understanding the intricate role of the cGAS–STING pathway in urologic malignancies.

## 4 CHARACTERISTICS OF TUMOURS

### 4.1 Cellular level

#### 4.1.1 Programmed cell death

A variety of programmed cell death mechanisms, including apoptosis, ferroptosis, autophagy and pyroptosis, play pivotal roles in tumour progression. Apoptosis is a specific programmed mechanism of cell death, featured by noninflammatory clearance of dying or damaged cells by adjacent cells.^42^ Apoptosis is orchestrated by enzymes known as caspases, which can be activated by the extrinsic and intrinsic pathways.^43^, ^44^ In the extrinsic pathway, death receptors interact with ligand, thereby activating caspases and facilitating the clearance of damaged cells.^43^ By contrast, the intrinsic apoptotic pathway involves cytochrome *C* release induced by mitochondrial outer membrane permeabilisation (MOMP), thus mediating caspase activation.^44^ Collectively, these two pathways converge on caspase activation. Importantly, apoptosis serves a crucial role in tumour regression. Impaired apoptosis, characterised by elevated anti‐apoptosis Bcl‐2 and reduced pro‐apoptotic Bax, triggers the development of chronic lymphocytic leukaemia,^45^ and neuroblastoma and glioblastoma.^46^ These findings underscore the contribution of impaired apoptosis in cancer progression.

Ferroptosis is driven by the buildup of lethal lipid peroxidation, leading to imbalanced redox homeostasis and disrupted cell metabolism.^47^ The defining feature of this iron‐dependent process the buildup of redox‐active Fe^2+^, ultimately resulting in lipid peroxidation that damages the cell membrane and triggers cell death.^48^ System Xc^−^ is one of the important components in ferroptosis.^49^ System Xc^−^ takes up extracellular Cys molecules to synthesise glutathione, sustaining glutathione peroxidase 4‐mediated detoxification of lipid hydroperoxides and thereby inhibiting ferroptosis.^49^ Importantly, overexpressed system Xc^−^ is associated with tumour development. The up‐regulated system Xc^−^ has been shown to suppress ferroptosis, thereby accelerating the development of PCa, melanoma and glioma.^50^, ^51^, ^52^ To summarise, ferroptosis suppression positions a pro‐tumourigenic role.

Autophagy is an essential catabolic process vital for maintaining cellular homeostasis. Its main function is to perform quality control on proteins and organelles within the cytoplasm. It manifests dual roles in tumourigenesis, serving as either a suppressor of tumour growth or a facilitator of tumour promotion. During the early stages, autophagy inhibits chronic tissue damage and prevents oncogenesis by clearing damaged proteins and organelles accumulated under stress conditions.^53^ In advanced stages, however, it supports tumour survival and growth by mitigating oxidative stress and removing harmful cellular debris, thereby facilitating the progression of benign lesions toward malignancy.^53^ Furthermore, autophagy enhances tumour invasiveness by promoting metastatic processes.^54^ Pyroptosis, an inflammatory form of programmed cell death, is marked by osmotic lysis, inflammatory cytokine release and pyroptotic cell death.^55^ Emerging evidence implicates that pyroptosis up‐regulates the expression of multiple proinflammatory cytokines and chemokines, highlighting its functional relevance in tumour immunomodulation.^56^

#### 4.1.2 Senescence

Cellular senescence denotes a persistent halt in the cell cycle that functions as a crucial anti‐tumour mechanism.^57^ An important hallmark of cellular senescence is the senescence‐associated secretory phenotype (SASP). This phenotype is marked by the sustained release of a diverse array of inflammatory mediators, including IL‐8, IL‐1α and IFN.^57^ These SASP components have the capability to orchestrate tumour microenvironment (TME) remodelling, leading to senescence and particularly establishing tumour‐suppressive niches. For example, SASP factors released by Th1 lymphocytes promote tumour senescence and simultaneously prevent tumour progression.^58^ Based on these studies, SASP serves as a central executor of senescence‐dependent tumour suppression. Multiple studies have elucidated that NF‐κB is recognised as a main regulatory factor for SASP components.^59^, ^60^ Disruption of NF‐κB results in cellular senescence escape, presenting the low expression of IL‐8 and IL‐6. Overall, the occurrence of cellular senescence can inhibit tumour progression, providing a novel strategy for targeting tumours.

#### 4.1.3 Mitochondrial calcium homeostasis

Cytosolic Ca^2+^ concentrations are typically maintained at around 100 nM through transport systems.^61^ To keep cytosolic Ca^2+^ on a relatively stable level, Ca^2+^ is actively transported to the ER, mitochondria or extracellular space via calcium channels or pumps.^61^ The ER, the largest intracellular Ca^2+^ reservoir, transports Ca^2+^ directly to mitochondria through specialised membrane contact, termed mitochondria‐associated ER membranes (MAMs).^62^ This process is initiated by the inositol 1,4,5‐trisphosphate (IP_3_) binding to IP_3_ receptors (IP_3_Rs) on the ER membrane, promoting the Ca^2+^ release.^62^, ^63^ Given the high permeability of the voltage‐dependent anion channel 2 (VDAC2) on the mitochondrial outer membrane, cGAS–STING pathway from the ER flows into mitochondria in large amounts through MAMs.^62^, ^63^ This enhanced level of Ca^2+^ transporting from the ER to mitochondria facilitates cancer cell death.^64^ For example, MAM‐localised proteins like promyelocytic leukaemia protein and phosphatase and tensin homologue evoke ER–mitochondria Ca^2+^ connection upon exposure to cancer therapeutic agents, thus promoting cell death.^65^, ^66^ These studies clarified that ER–mitochondria Ca^2+^ influx leads to tumour suppression and represents a potential target for tumour treatment.

#### 4.1.4 Tryptophan metabolism

The aryl hydrocarbon receptor (AhR), a ligand‐activated transcription factor, is activated by diverse metabolic and dietary signals.^67^ The crosstalk between AhR activation and tryptophan (Trp) metabolism, especially via the kynurenine (Kyn) pathway, constitutes a pivotal regulatory axis in tumour immune evasion.^67^ Mechanistically, Trp is predominantly catabolised to Kyn by indoleamine 2,3‐dioxygenase 1 (IDO1).^68^ Functioning as an endogenous AhR ligand, Kyn binds AhR and mediates its nuclear translocation.^68^ Within the nucleus, AhR dimerises, driving pro‐tumourigenic cellular phenotypes.^69^ Critically, the Kyn–AhR axis exerts profound immunosuppressive effects: it promotes the differentiation and functional activity of regulatory T cells, induces CD8^+^ cytotoxic T cell apoptosis or dysfunction and facilitates recruitment and polarisation of tumour‐associated macrophages (TAMs) toward an immunosuppressive phenotype.^70^, ^71^ Consequently, sustained activation of this IDO1/Kyn/AhR pathway potently facilitates tumour immune escape.^71^ Therapeutic targeting of this metabolic–immune checkpoint, particularly through IDO1 inhibition or Trp restriction, represents a compelling anti‐tumour strategy.

### 4.2 Tissue level

#### 4.2.1 Regulate the TME

The TME serves as a critical niche facilitating cancer onset and progression, encompassing noncancer cells and other noncellular components. Noncancer cells encompass a variety of cell types, including cancer‐associated fibroblasts (CAFs), TAMs and other cellular components, while noncellular components include extracellular matrix and various soluble molecules, such as TGF‐β and IL‐6. These components maintain TME homeostasis and play essential roles in tumour progression. Among these components, CAFs are the most abundant population in TME.^72^ Solute carrier family 14 member 1 (SLC14A1), a urea transporter highly expressed in CAFs, remodels cell adhesion mechanisms, thereby promoting cancer stemness and chemoresistance.^73^, ^74^ TAMs polarise into two major subtypes: M1 macrophages exhibiting anti‐tumourigenic effects through elevated inflammation, and M2 macrophages facilitating tumour progression via promoting cellular proliferation, immunosuppression and angiogenesis.^75^ TGF‐β, a potent pro‐tumourigenic factor, drives CAF activation and M2 TAM polarisation.^73^, ^76^ IL‐6 selectively modulates T cell recruitment^77^ and promotes CD206+ M2 macrophage polarisation by inhibiting caspase‐8 cleavage and enhancing autophagy,^78^ collectively mediating tumour recurrence.^79^ Consequently, targeting specific TME components represents a therapeutic strategy for tumours.

### 4.3 Molecular level

#### 4.3.1 DNA damage

Among multiple types of DNA damage, double‐strand breaks (DSBs) are the most serious form of DNA damage, with insufficient repair directly leading to the occurrence of breast cancer, PCa and pancreatic cancer.^80^ Paradoxically, emerging evidence indicates that DNA damage activates the innate immune pathway of STING, thereby eliciting potent anti‐tumour immunity. cGAS detects exogenous viral DNA in the cytoplasm and then activates STING, thereby mediating IFN signalling.^81^ Importantly, recent studies have delineated a noncanonical STING (NC‐STING) activation independent of cGAS. Following DNA damage, various DNA repair proteins, including ataxia–telangiectasia mutated kinase, poly (ADP‐ribose) polymerase 1 (PARP1) and IFN‐inducible protein 16 (IFI16), play a direct role in activating STING. This activation promotes the recruitment of tumour suppressor gene p53.^82^ DNA damage leads to a dual effect, providing more possibilities for targeted therapy of tumours.

## 5 DUAL ROLES OF THE cGAS–STING PATHWAY IN TUMOUR PROGRESSION

**FIGURE 2:**
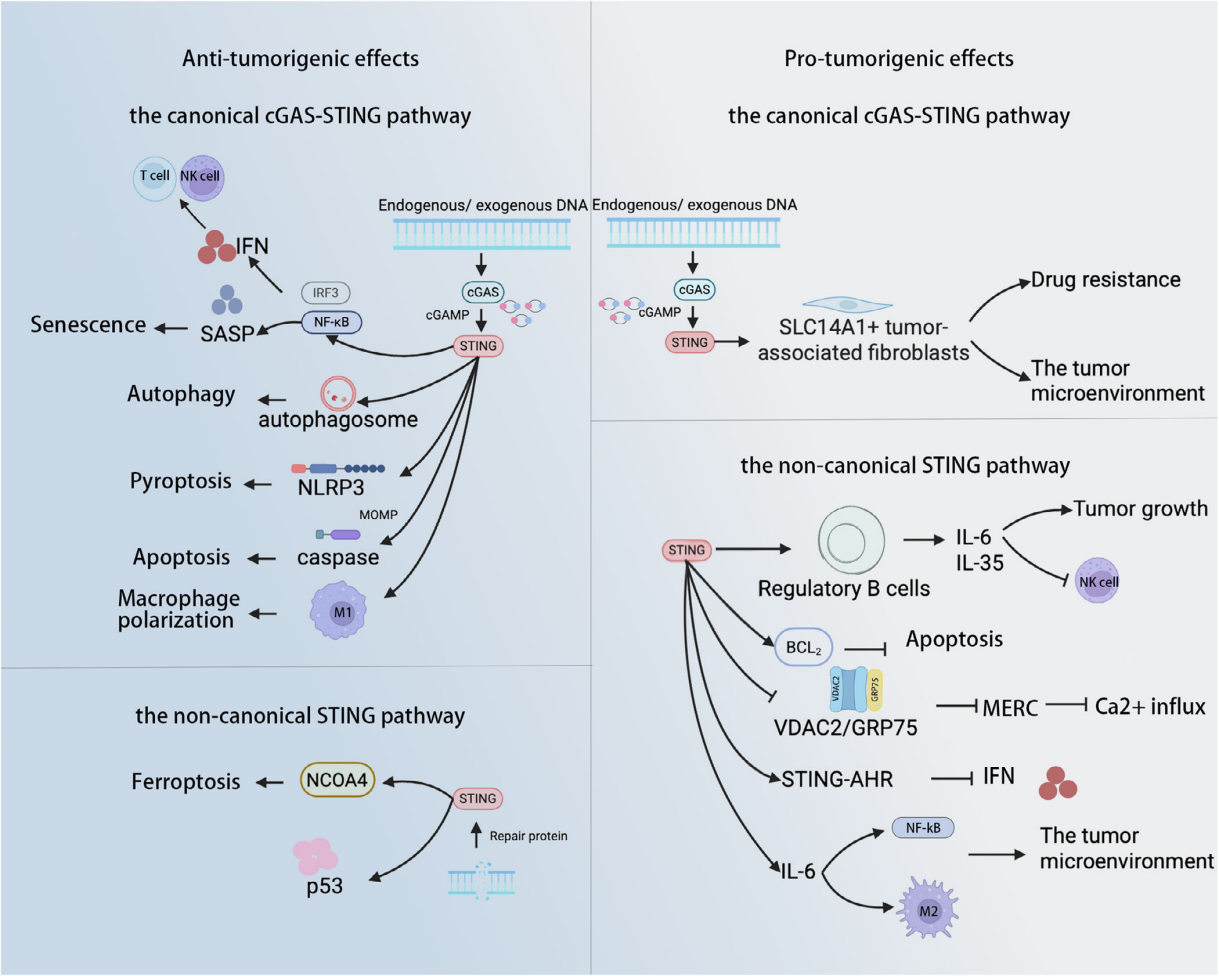
Dual roles of the cGAS–STING pathway. The cGAS–STING pathway plays a complex dual role in urological malignancies. Its canonical activation can recruit CD8+ T cells and trigger various tumour‐suppressive cell death mechanisms, such as ferroptosis, apoptosis, autophagy and pyroptosis. Conversely, persistent pathway activation, NC‐STING signalling or specific downstream effector engagement may promote tumourigenesis. For instance, canonical cGAS–STING activation can shape TME by up‐regulating CAFs. Moreover, NC‐STING drives TME remodelling through Breg cell expansion, disruption of mitochondrial calcium homeostasis and metabolic interference, collectively facilitating cancer progression.

The cGAS–STING pathway exhibits a complex, context‐dependent duality in urological malignancies (Figure 2). Canonical pathway activation recruits CD8^+^ T cells, triggers pro‐inflammatory cytokine release and promotes tumour‐suppressive cell death mechanisms including ferroptosis and apoptosis, thereby establishing anti‐tumour immunity.^83^, ^84^ Conversely, persistent cGAS–STING activation, NC‐STING signalling or engagement of specific downstream effectors can drive pro‐tumourigenic outcomes. NC‐STING pathway promotes regulatory B (Breg) cell expansion and NF‐κB expression, reprogramming TAMs and the TME to facilitate tumour progression.^85^, ^86^ This profound functional dichotomy is intricately governed by factors, including different cell types, stimulating factors, and the inherent characteristics of tumours. Consequently, deciphering the specific regulatory modes and effector networks of this pathway in the three major urological cancers—PCa, RCC and BCa—is a critical prerequisite for developing effective targeted therapies.

## 6 REGULATORY ROLE OF cGAS–STING‐RELATED PREPARATIONS IN CASTRATION‐RESISTANT PCa

PCas manifest as an immunologically ‘cold’ cancer, characterised by insufficient infiltration of immune cells in TME, resulting in limited efficacy for targeted therapy or immunotherapy.^87^ Evidence suggests that the cCAS–STING pathway enhances immune cell infiltration and may be a therapeutic target for PCa therapy. The classical pathway promotes apoptosis,^84^ ferroptosis^83^ and inflammatory response,^40^ inhibiting PCa progression. However, the NC‐STING pathway exhibits a paradoxical function in castration‐resistant PCa (CRPC). On one hand, the NC‐STING signalling pathway can up‐regulate ferroptosis by increasing mitochondrial membrane density and the absence of cristae, inhibiting PCa progression.^83^ On the other hand, it facilitates the progression of PCa by regulating Breg cells,^86^ IL‐6 and NF‐KB^85^ in the TME. These studies provide a foundational rationale for devising novel preclinical strategies targeting this cGAS–STING pathway.

### 6.1 Modulating cell death

Accumulating evidence indicates that promoting apoptosis and ferroptosis represents a therapeutic strategy for tumour suppression. Emerging evidence reveals crosstalk between apoptosis and cGAS–STING signalling. Specifically, this pathway regulates MOMP, a crucial step in the intrinsic activation pathway of apoptosis, thus amplifying caspase‐dependent cell death.^84^ Moreover, the pro‐apoptosis protein Bax contributes to mtDNA release, appealing cGAS accumulation and subsequently driving STING pathway activation.^88^ These processes create a self‐reinforcing loop: the cGAS–STING pathway facilitates apoptosis through MOMP activation, while apoptosis‐driven mtDNA release potentiates the cGAS–STING activation. Overall, this cGAS–STING‐apoptosis axis displays potential targets that overcome tumour immune evasion.

Ferroptosis acts as a pivotal mechanism in inhibiting tumour progression. The activation of both NC‐STING signalling and the classical cGAS–STING pathway up‐regulates ferroptosis. Mechanistically, the C‐terminal region of STING binds to the N‐terminal coiled‐coil domain of the cytoplasmic nuclear receptor coactivator 4 (NCOA4), mediating the liberation of free ferrous iron and initiating a cascade of lipid peroxidation that drives cell death.^83^ STING agonist stimulation induces mitochondrial vacuolisation, increased membrane density and loss of cristae—hallmarks of ferroptosis.^83^ These results indicate that the STING pathway promotes ferroptosis, thereby dampening tumour development. However, recent studies demonstrate that cGAS suppresses ferroptosis and mitochondrial ROS accumulation by binding to DRP1 (dynamin‐related protein 1) on mitochondrial membranes.^89^ Collectively, the classical pathway and cGAS/DRP1 axis provide dual regulatory perspectives on ferroptosis, offering novel therapeutic strategies for modulating cell death in tumours.

The cGAS–STING pathway exerts a key modulator of both autophagy and pyroptosis. It primarily triggers noncanonical autophagy to facilitate the clearance of cytosolic DNA. Upon binding of cGAMP, STING detaches from the ER and enters vesicles modified by COP‐II, which subsequently form ERGIC. The ERGIC leads to autophagosome formation and degradation of cytoplasmic DNA.^90^ Moreover, this pathway promotes pyroptosis and inflammatory responses in an NLRP3 inflammasome‐dependent manner.^91^ Collectively, these mechanisms highlight the pivotal involvement of cGAS–STING pathway in modulating programmed cell death, offering a valuable therapeutic target to counteract tumour immune evasion.

**FIGURE 3:**
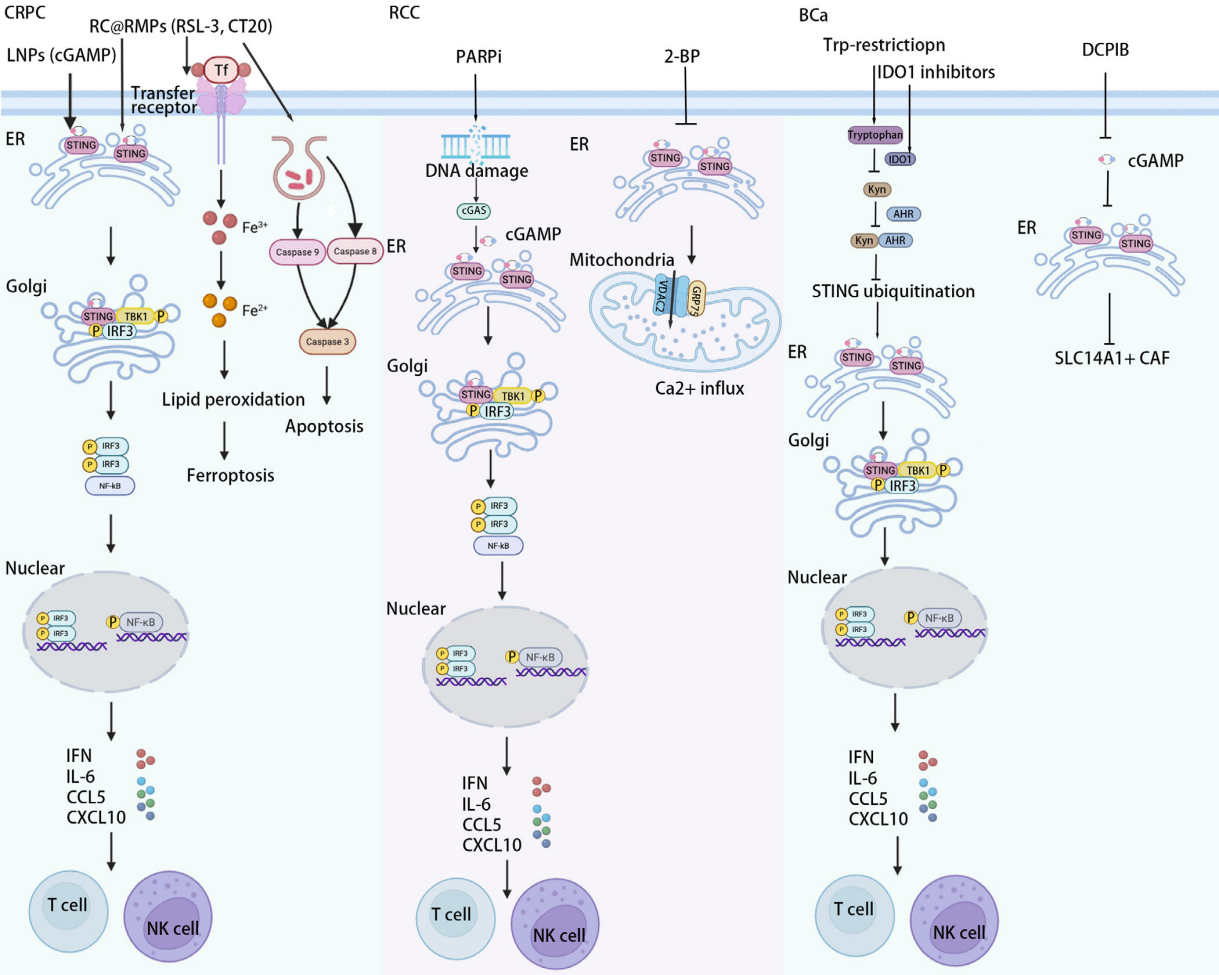
Treatment approaches targeting the cGAS–STING pathway in urological malignancies. Based on the dual regulatory role of the cGAS–STING pathway in urological malignancies, we have systematically summarised relevant therapeutic strategies. In prostate cancer, various carriers (such as RC@RMPs and LNPs) activate the cGAS–STING pathway, promote interferon release and subsequently activate CD8⁺ T cells and NK cells to enhance anti‐tumour immunity. In RCC, PARPi induces the cGAS–STING signalling via DNA damage, triggering an immune response. Additionally, 2‐BP inhibits palmitoylation of the STING protein, promotes the interaction between VDAC2 and GRP75, enhances calcium influx, and thereby induces tumour cell death. In BCa, metabolic intervention promotes the accumulation of the STING protein, activating its downstream signalling pathway. The inhibitor DCPIB suppresses the proportion of STING‐mediated SLC14A1⁺ CAFs, thereby inhibiting tumour progression.

Recent advancements have highlighted that RMPs, small vesicles released by irradiated tumour cells, can stimulate relatively low levels of ferroptosis.^92^ Specifically, RMPs boost ferroptosis level through activating the cGAS–STING pathway^93^ (Figure 3). RMPs also serve as precision drug carriers,^94^ capable of delivering the ferroptosis inducer RSL‐3 as well as the apoptosis inducer CT20 peptide (CT20p) to target cells, thus initiating ferroptosis and apoptosis.^93^ This combination, referred to as RC@RMPs, elicits a significantly strong ferroptosis and apoptosis response. In addition to directly triggering cell death, RMPs can also reprogram the TME by polarising TAM toward M1 phenotypes.^92^ The increased polarisation of M1 macrophages can promote the occurrence of inflammation, thereby improving the anti‐tumour immune response and further inhibiting PCa development.^92^ Overall, RMPs facilitate ferroptosis and apoptosis via up‐regulating cGAS–STING signalling. This anti‐tumour synergistic effects of enhancing cell death and remodelling the TME position RMPs as a pivotal avenue for precision oncology.

### 6.2 Enhancing CD8^+^ T cells and B cells

A wide range of mature CD8^+^ T cells is the key factor in dampening CRPC development, due in part to mediate a potent anti‐tumour response.^40^ cGAS–STING pathway emerges as a critical regulator of CD8^+^ T cell maturation through triggering IFN secretion. This immunostimulatory axis can be amplified by LNPs.^40^ LNPs, loaded with robust cGAMP, are delivered to perivascular to initiate this pathway, thus enhancing CD8^+^ T cell maturation and elevating the number of CD4^+^ T cells and NK cells in mouse PCa^40^ (Figure 3). These elevated mature CD8^+^ T cells prolong the therapeutic efficacy of androgen deprivation therapy (ADT) and postpone the CRPC development. Furthermore, epigenetic modulation provides complementary therapeutic leverage. Enhancer of zeste homolog‐2 (EZH2) overexpression is related to PCa progression.^95^ EZH2 inhibition initiates an RNA–STING–ISG pathway to an increase in CD8^+^ T cell levels, hence retarding CRPC progression.^96^ Collectively, LNPs and EZH2 amplify cGAS–STING pathway activation and subsequently induce an expansion of numerous mature CD8^+^ T cells, suppressing the development of CRPC. This pathway provides a viable avenue for converting immunological ‘cold’ tumours to ‘hot’ tumours.

However, an emerging study has revealed that STING agonist monotherapy induces significant tumour resistance and can promote tumour immune evasion and metastasis.^97^ Breg cells, known for their immunosuppressive properties, contribute to immune tolerance in cancer.^98^ STING agonists up‐regulate the production of IL‐35 and IL‐10 in Breg cells, which in turn drives tumour growth.^86^ For instance, in cGAMP‐treated pancreatic ductal adenocarcinoma mouse models, Breg cell‐derived IL‐35 and IL‐10 are markedly elevated, and their levels correlate positively with tumour weight.^86^ Concurrently, Breg cell‐derived IL‐35 suppresses the anti‐cancer activity of NK cells.^86^ These pro‐tumourigenic functions of STING activation underscore the promise of targeting IL‐35 as a novel strategy to inhibit tumour growth.

### 6.3 Modulating NF‐KB expression

It is noteworthy that the cGAS–STING–NF‐κB signalling axis exerts a biphasic role in tumours. In one aspect, it inhibits tumour development by enhancing SASP release.^99^ Moreover, it enhances immunosuppressive activity, thereby potentially fostering tumour advancement.^100^ Studies have demonstrated that stimulation of this signalling accelerates tumour progression in mouse models of PCa. Additionally, the NC‐STING pathway critically promotes CRPC progression.^85^ In PCa driven by SPOP mutations (SPOPmut), impaired degradation leads to accumulation of STING1 protein, which activates the NC‐STING pathway. This pathway activates NF‐κB expression and promotes M2 macrophage polarisation through IL‐6, collectively facilitating CRPC progression.^85^ Furthermore, NC‐STING signalling enhances the anti‐apoptotic protein BCL‐2 expression, further contributing to tumour progression.^85^ Collectively, these results highlight the central influence of the TME in CRPC. This pathway emerges as a pivotal role in driving CRPC progression by orchestrating multiple cytokine networks within the TME, making it a promising target.

## 7 DUAL REGULATORY ROLE OF STING AGONISTS/INHIBITORS IN RCC

The cGAS–STING pathway activation exhibits a biphasic regulatory function in RCC progression. The classical pathway exerts anti‐tumour effects. DNA damage activates cGAS–STING, inducing up‐regulated inflammatory factors, including IFN and TNF‐α, thereby inhibiting RCC progression.^12^ Additionally, cGAS‐independent NC‐STING activation demonstrates tumour‐suppressive functions.^82^ Following DNA damage, repair proteins (including PARP inhibition and IFI16) directly activate STING, facilitating recruitment of the tumour suppressor p53.^82^ Paradoxically, NC‐STING also exhibits tumour‐promoting activity. It accelerates RCC progression by modulating intracellular calcium homeostasis.^10^ The duality of STING signalling underscores its complexity in RCC and highlights its therapeutic potential.

### 7.1 Facilitate DNA damage

DNA damage exhibits anti‐tumour effects partially via activating the classical pathway, as previously proposed. PARP1, a prevalent nuclear protein, is involved in single‐strand breaks (SSBs) repair, maintaining genomic stability.^11^ PARPi, preventing the catalytic function of PARP1, leads to the generation and buildup of DSBs.^101^ Unrepaired DSBs initiate the innate immune pathway of cGAS–STING, subsequently up‐regulating IFN, TNF‐α and other inflammatory factors^12^ (Figure 3). It is noteworthy that the therapeutic efficacy of PARPi is further amplified in SETD2‐deficient contexts. SETD2, encoding a histone H3 lysine 36 (H3K36) trimethylase, participates in DNA damage repair.^102^ In SETD2‐deficient cells, the ability of DNA repair has been down‐regulated, leading to DSBs accumulation. This augmented DNA damage robustly initiates the cGAS–STING pathway, thus retarding RCC progression.^102^, ^103^ In summary, the cGAS–STING pathway activated by DNA damage establishes a potential approach for RCC treatment.

### 7.2 Inhibit STING palmitoyl‐transferases

**FIGURE 4:**
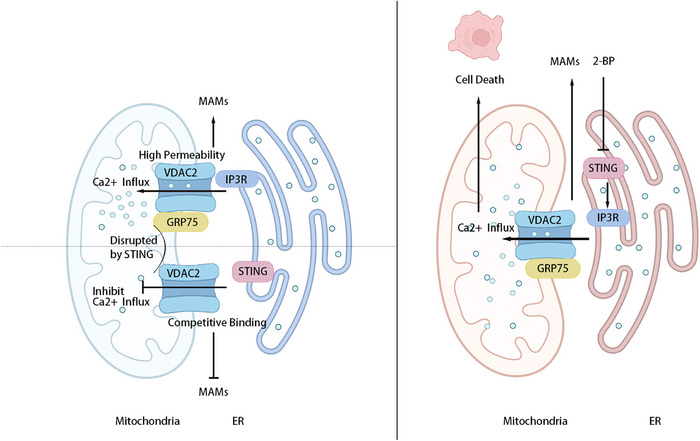
Mechanistic overview of cGAS–STING‐mediated calcium homeostasis regulation. The ER, serving as the primary intracellular Ca^2^⁺ storage site, directly transfers Ca^2^⁺ to mitochondria via MAMs. Specifically, IP_3_ binds to IP_3_R on ER membrane, facilitating Ca^2^⁺ release. VDAC2 on the mitochondrial outer membrane exhibits high permeability and promotes massive Ca^2^⁺ influx into mitochondria upon binding to GRP75, which bridges VDAC2 to IP_3_Rs. STING activation suppresses this Ca^2^⁺ influx. Specifically, STING interacts with VDAC2 on the mitochondrial outer membrane, disrupting the VDAC2–GRP75 interaction. This STING–VDAC2 association destabilises MAMs, thereby restricting Ca^2^⁺ transfer. Conversely, STING inhibition restores GRP75–VDAC2 binding, enhancing MAMs integrity and increasing mitochondrial Ca^2^⁺ flux. These findings suggest a competitive binding mechanism whereby STING may displace GRP75 to associate with VDAC2.

Several pieces of evidence link the cGAS–STING pathway to mitochondrial calcium homeostasis. In fibroblasts and T cells, the absence of STING results in enhanced Ca^2+^ influx.^104^ Specifically, STING depletion significantly augments mitochondria‐endoplasmic reticulum contact (MERC), thereby promoting Ca^2+^ influx.^105^ Mechanistically, STING interacts with VDAC2 on the outer mitochondrial membrane, thereby disturbing its interaction with glucose‐regulated protein 75 (GRP75), a critical MERC component that bridges VDAC2 and IP_3_R^10^ (Figure 4). This STING–VDAC2 interaction breaks MERC and subsequently limits the Ca^2+^ transfer.^10^ These studies indicate that STING may act as a competitor to GRP75 in its interaction with VDAC2. Enhancing the connection between GRP75 and VDAC2 mediates increased Ca^2+^ transfer from ER to mitochondria, thus improving sensitivity to tumour treatment and enhancing clinical outcomes. These findings position STING as a pivotal role in mitochondrial calcium homeostasis, with its inhibition offering a novel avenue to disrupt tumour development through calcium overload.

Palmitoylation is essential for STING function.^26^ Mutation of Cys88 and Cys91 residues markedly reduces STING palmitoylation, which severely impairs IFN‐I response.^26^ Moreover, these two Cys residues are also critical for the interaction between STING and VDAC2.^10^ Specifically, STING–C88A/C91A mutations disturb STING–VDAC2 binding through reducing STING palmitoylation.^10^ These findings underscore the essential role of palmitoylation in maintaining STING–VDAC2 complex integrity. Therapeutically, 2‐BP exhibits an inhibitory capacity toward STING palmitoylation and subsequently elevates VDAC2/GRP75/IP3R complex‐mediated mitochondrial Ca^2+^ influx^10^, ^106^ (Figure 3). This amplified mitochondrial Ca^2+^ influx exhibits tumour‐suppressive effects, significantly inhibiting the growth of RCC makers, including 786‐o, UMRC6 and RCC10, and reducing the weight of RCC (*p* = .01366).^10^ In conclusion, STING palmitoylation has a pivotal role in controlling its interaction with VDAC2 and mitochondrial Ca^2+^ homeostasis. 2‐BP disturbs the interaction, triggering an anti‐tumour response. These studies highlight STING palmitoylation as a pivotal target in RCC treatment.

## 8 DUAL REGULATORY ROLE OF STING IN BCa

BCa management primarily relies on cisplatin‐based neoadjuvant therapy and immunotherapy, yet suboptimal responses in a patient subset necessitate strategies to overcome resistance. Specifically, Trp suppresses the STING pathway via Kyn–AhR axis to modulate IFN production in BCa, potentially contributing to poor neoadjuvant chemotherapy responses in some patients.^107^ Intriguingly, this pathway also exhibits tumour‐enhancing functions in BCa. It enhances cancer cell stemness and chemoresistance by up‐regulating SLC14A1^+^ CAFs.^107^ These insights reveal novel strategies to overcome chemoresistance.

### 8.1 Targeting Kyn–AhR–STING axis

Cisplatin‐based treatment remains the standard care of BCa patients. However, a subset of patients exhibits suboptimal response to chemotherapy and immunotherapy, which is related to AhR overexpression. Elevated AhR expression in BCa promotes STING ubiquitination. Specifically, AhR, upon binding endogenous ligands (e.g., Trp and its metabolites) or exogenous ligands (e.g., aromatic hydrocarbons in tobacco and hair dyes), mediates ubiquitination of STING at lysine 236 (K236), reducing STING protein stability.^107^ Compromised STING function down‐regulates IFN expression, driving immune resistance.^107^ Therefore, the overexpression of AhR mediates therapeutic resistance by suppressing the STING pathway. Importantly, Trp regulates the expression of this pathway through binding AhR. For example, in a mouse model that is constructed by the murine BCa cells, Trp‐restricted diet enhances STING‐dependent IFN production with robust CD8^+^ T cells infiltration.^107^ Thus, inhibiting Trp and its metabolite Kyn may be a potential and promising strategy to enhance NC‐STING pathway (Figure 3). As Trp metabolism is predominantly catalysed by IDO1 to form Kyn,^108^ IDO1 inhibitors reduce Kyn, thereby inhibiting the Kyn–AhR–STING axis.^107^ This approach overcomes the immunosuppression and enhances sensitivity to chemotherapy. To conclude, this Kyn–AhR axis serves as a critical regulator of STING‐mediated tumour‐suppressive effects. Targeting Trp and its metabolite Kyn to restore STING‐mediated anti‐tumour immunity offers a viable approach to sensitise BCa to conventional therapies.

### 8.2 Inhibit SLC14A1

SLC14A1 enhances the stemness and resistance to chemotherapy in tumour cells by secreting a variety of growth factors.^74^ Targeting SLC14A1 may be a promising avenue in enhancing the sensitivity of BCa chemotherapy. SLC14A1 dynamics can be modulated by cGAS–STING pathway activation. Evidence indicates that this pathway elevates IFN production, increasing the fraction of SLC14A1^+^ CAFs and their transcriptional activities in BCa patients.^74^ In alignment with this, genetic ablation (STING−/−) or pharmacological inhibition of STING exhibited a decreased SLC14A1^+^ CAF ratio in a BCa model.^74^ These investigations suggest that STING activation enhances the level of SLC14A1, thus promoting BCa progression. Targeting the cGAS–STING–SLC14A1 axis represents an innovative therapeutic approach for BCa therapy. For instance, the inhibitor DCPIB suppresses cGAMP transport, inhibiting STING activation, SLC14A1 expression and IFN production, thereby attenuating SLC14A1+ CAF‐mediated tumourigenesis (Figure 3).^74^ Overall, targeting the cGAS–STING–SLC14A1 axis provides potential methods for improving the therapeutic effect of BCa treatment.

## 9 CLINICAL TRANSLATION CHALLENGES AND EMERGING THERAPEUTIC STRATEGIES

### 9.1 Sexual dimorphism in urologic malignancies

The incidence of urologic malignancies is markedly more prevalent in men compared with women, with BCa and RCC occurring at approximately 3.9 times and 2 times the rates in women, respectively.^1^ This disparity may be influenced by sex hormones and their receptors. Overexpression of the androgen receptor (AR) is an established driver in BCa and CRPC, while ADT suppresses proliferation in BCa cell lines.^109^ In contrast, oestrogen signalling, mediated by Erα, appears to suppress BCa pathogenesis.^110^ Similarly, AR‐siRNA knockdown and oestrogen treatment inhibit proliferation, migration and invasion while promoting cell death in RCC models.^111^ Critically, AR signalling closely interacts with the cGAS–STING pathway. Loss of AR induces dsDNA breaks and leakage, thereby activating cGAS–STING pathway.^112^ Thus, enhanced AR activity in males may suppress cGAS–STING‐mediated innate immunity, promoting tumour immune evasion and therapy resistance. Targeting this AR–STING axis presents a high promise for urologic cancers.

Besides the influence of sex hormones and their receptors, genetic factors associated with the X chromosome may also play a significant role in the sex‐based disparities observed in urologic tumours. Females are defined by an XX chromosome pair, while males are defined by an XY chromosome pair. A proportion of X‐linked genes escape X‐chromosome inactivation, which leads to elevated expression levels of certain immune‐related genes in females, such as Toll‐like receptor 7 (TLR7) and the histone demethylase KDM6A. ^113^ This genetic background may contribute to differences in anti‐tumour immune responses between the sexes. Specifically, biallelic expression of TLR7 can induce greater production of IFN‐α, thereby enhancing the early recognition and clearance of tumour‐associated antigens in females. ^114^ Furthermore, the X‐linked gene KDM6A regulates the p53 signalling pathway, mediating sex‐based differences in tumourigenesis and progression.^115^ In summary, the combined effects of weaker AR signalling and X chromosome‐linked genetic mechanisms help explain the sex‐based disparity in the incidence of urologic tumours. Beyond these sex‐specific regulatory mechanisms, the functional output of the cGAS–STING pathway is precisely determined by a diverse array of factors that govern its bidirectional signalling.

### 9.2 Influencing factors of bidirectional cGAS–STING signalling

The function of the cGAS–STING signalling pathway is highly context‐dependent, playing a dichotomous function in oncogenesis that is co‐determined by a multitude of factors. This context can be deciphered through three fundamental layers of regulation. First, this pathway exhibits cell‐type specificity. The canonical pathway typically exerts anti‐tumour effects by activating CD8⁺ T cells and inducing IFN production.^40^ Conversely, NC‐STING signalling in specific immune cells can drive pro‐tumourigenic outcomes. For instance, in PCa, NC‐STING signalling up‐regulates the production of IL‐35 and IL‐10 by Breg cells, which leads to the inhibition of NK and CD8⁺ T cells, ultimately fostering immune escape.^86^ Beyond immune cells, the pathway's role is equally complex in tumour cells and TME components. In cancer cells, canonical signalling can promote cell death,^40^ whereas NC‐STING signalling activates NF‐κB and up‐regulates anti‐apoptotic proteins like BCL‐2,^85^ thereby accelerating disease progression. Similarly, in TAMs, the pathway can dictate polarisation fate: RMPs can promote anti‐tumour M1 polarisation,^92^ while in SPOP‐mutant PCa, NC‐STING signalling induces IL‐6 to drive pro‐tumour M2 polarisation.^85^ Therefore, precise cellular targeting of STING modulators is paramount to harnessing its anti‐tumour effects.

Different stimulus signals determine the different ‘directions’ of this pathway. Nuclear DNA damage, mtDNA release or DNA mediated by bacteria or viruses facilitates the canonical cGAS–STING pathway, triggering IFN expression and anti‐tumour immunity.^34^ Nevertheless, the activation of NC‐STING is linked to genetic mutations, metabolic rewiring and pharmacological effects. For example, in SPOP‐mutant PCa, SPOP loss (F102C/F133V) leads to STING protein stabilisation and accumulation, preferentially activating the NC‐STING–NF‐κB pathway, thereby promoting tumour growth.^85^ In BCa, overexpression of AhR facilitates STING ubiquitination and degradation, suppressing the entire STING–TBK1–IRF3–IFN axis and leading to immunotherapy resistance.^107^ In RCC, 2‐BP suppresses STING palmitoylation, thereby increasing the mitochondrial calcium influx mediated by the VDAC2/GRP75/IP3R complex and promoting cell death.^10^ Thus, genetic mutations, metabolic rewiring and pharmacological effects collectively create a decisive context that steers STING signalling toward divergent downstream outcomes.

Finally, the intrinsic characteristics of tumours set the foundation for the function of cGAS–STING pathway. The activation of this signalling differs dramatically between immunologically ‘cold’ (T cell‐deficient) and ‘hot’ (T cell‐inflamed) tumours. Cold tumours exhibit a lack of T cells and suppression of this pathway, thereby allowing immune tolerance and tumour‐promoting effects to predominate. Additionally, the metabolic state of cancer cells can directly inhibit this pathway. As exemplified in BCa, AhR activation by Trp metabolites inhibits STING, creating an immunosuppressive environment.^107^ Consequently, an inhibitory immune and metabolic environment may lead to a low expression of the cGAS–STING signalling pathway.

### 9.3 Resistance and adverse reaction of cGAS–STING signalling

The cGAS–STING pathway exhibits a paradoxical role in oncology, serving as both a critical regulatory factor for anti‐cancer immunity and a crucial mechanism underlying therapy resistance. Anticancer agents such as PARPi and cisplatin, which can damage DNA, can activate this pathway to suppress cancer progression. However, excessive activation of the pathway can conversely mediate acquired drug resistance. Specifically, the cGAS–STING pathway promotes tumour resistance through downstream TBK1–IRF3/p65 NF‐κB signalling.^116^ TBK1 has been recognised as a pivotal target in this process. For example, TBK1 inhibitors can reverse osimertinib‐induced STING‐dependent resistance and cell migration.^116^ Furthermore, systemic administration of STING agonists triggers various adverse effects, including systemic inflammatory responses and cytokine release syndrome.^117^ Studies demonstrate that overactivation of the cGAS–STING pathway in macrophages and monocytes is related to sepsis.^117^ In summary, cGAS–STING activation can not only facilitate tumour immune evasion but also induce a hyperinflammatory state.

Improving the adverse effects related to the cGAS–STING pathway represents an urgent need in the field. Rational combination therapies present a potential approach to circumvent resistance. The combination of STING agonists with TBK1 inhibitors can effectively counter STING‐dependent resistance and cell migration.^116^ Moreover, compared with systemic activation, cell‐specific targeting or localised activation of the pathway reduces adverse effects and improves therapeutic efficacy. Utilising nanocarriers (e.g., LNPs) or engineered cellular vesicles (e.g., RMPs) for tumour‐targeted delivery or local administration significantly mitigates systemic inflammatory reactions while maximising antigen presentation and cytotoxic T lymphocyte infiltration.^40^, ^92^

### 9.4 Combined therapy with STING agonists

Beyond mitigating resistance and toxicity, enhancing clinical efficacy remains a central focus. Combining STING agonists with immune checkpoint inhibitors (ICIs) holds substantial promise for cancer therapy. Activation of the cGAS–STING pathway enhances the infiltration and function of CD8⁺ T and NK cells,^118^ while tumour‐expressed PD‐L1 promotes immune evasion by inhibiting cytotoxic T cell activity.^119^ Together, these agents synergistically improve anti‐cancer immune responses and reverse immune resistance. Furthermore, the cGAS–STING pathway also enhances the efficacy of radiotherapy and chemotherapy by up‐regulating the expression of IFN.^107^, ^120^ Therefore, the combination of STING agonists with immunotherapy and radiochemotherapy is an effective treatment strategy for inhibiting tumour progression.

It is noteworthy that the interaction between lentiviral vectors (LVs) and the cGAS–STING pathway offers unique advantages for gene therapy in BCa. Recognition of the DNA encapsulated in LVs by cGAS triggers the cGAS–STING signalling pathway, ultimately inducing IFN expression.^121^ Furthermore, LVs enable the long‐term stable expression of IFN. Studies have demonstrated that LV‐mediated IFN (LV‐IFN) synergistically induces tumour cell death in BCa models by triggering apoptosis and ER stress, significantly improving the survival rate in these models.^122^ This indicates that LVs can synergistically exert anti‐tumour effects by engaging both immunotherapeutic and gene therapeutic mechanisms.

Currently, clinical trials evaluating STING agonists (either as monotherapy or in combination with ICIs, radiotherapy or chemotherapy) for the treatment of solid tumours such as BCa, RCC and PCa have been extensively initiated (Table 1). In contrast, the advancement of STING‐targeted inhibitory agents remains in the preclinical stage. In conclusion, rationally designed combination strategies are crucial to addressing the challenges of STING‐targeted therapy. Achieving precise and controllable regulation of this pathway may ultimately harness its potent immunomodulatory capacity for sustained clinical benefit (Table 2).

**TABLE 1:** Clinical trials with STING agonists.

Agent	NCT code	Type of cancer	Phase	NCT trial status
ASA404	NCT00111618	Hormone refractory metastatic prostate cancer	2	Completed
CRD3874‐SI	NCT06021626	Sarcoma and merkel cell carcinoma	1	Recruiting
E7766	NCT04109092 (INPUT‐102)	NMIUC, BCG‐unresponsive	1	Withdrawn
E7766	NCT04144140	Advanced solid tumours or lymphomas	1/1b	Terminated
GSK3745417				
Dostarlimab	NCT03843359	Refractory/relapsed solid tumours	1	Active, not recruiting
IMSA101				
PULSAR‐ICI (pembrolizumab/nivolumab)	NCT05846646	RCC and NSCLC	2	Terminated
IMSA101	NCT06601296	Metastatic kidney cancer	2	Recruiting
IMSA101				
PULSAR‐ICI (pembrolizumab/nivolumab)	NCT05846659	Oligoprogressive solid tumour malignancies after prior anti‐cancer therapy	2	Terminated
KL340399	NCT05549804	Advanced solid tumours	1	Recruiting
MIW815(ADU‐S100)				
PD‐1 checkpoint inhibitor PDR001	NCT03172936	Advanced/metastatic solid tumours or lymphomas	1b	Terminated
ONM‐501				
Cemiplimab	NCT06022029	BCa and other solid tumours	1	Recruiting
SNX281				
Pembrolizumab	NCT04609579	Advanced solid tumours and lymphomas	1	Terminated
TAK‐500				
Pembrolizumab	NCT05070247	Kidney cancer and other solid tumours	1/2	Terminated
TXN10128				
Irinotecan or paclitaxel	NCT05978492	Locally advanced (unresectable) or metastatic solid tumours	1	Recruiting
Vadimezan (DMXAA, ASA404, AS1404)				
Docetaxel	NCT01071928 (GU09‐144)	Advanced urothelial cancer, 2ndline	2	Withdrawn
Vadimezan (DMXAA, ASA404, AS1404)	NCT01278758	Advanced solid tumours, impaired renal function	1	Terminated

*Note*: These data were obtained from https://clinicaltrials.gov/.

Abbreviations: PULSAR, personalised ultra‐hypofractionated stereotactic adaptive radiotherapy; ICI, immune checkpoint inhibitor; RCC, renal cell carcinoma; NSCLC, non‐small cell lung cancer; BCG, Bacillus Calmette–Guerin; NMIUC, nonmuscle invasive urothelial cancer.

**TABLE 2:** A summary table of drugs, targets and functions related to the cGAS–STING pathway.

Drug/name	Technology	Target	Model	Associated pathway	Regulated biochemical component	Function	References
STING agonists (ADU‐S100)	CRISPR–Cas9	STING1	Septic mice model	cGAS–STING–NCOA4	TBK1, GPX4, ferritin heavy chain, ferroportin	STING promotes ferroptosis and inflammation.	^83^
	Lentivirus‐delivered CRISPR–Cas9/shRNA	cGAS	Hepatocellular carcinoma cells and xenograft mouse models	cGAS–DRP1 axis	Glutathione, lipid peroxidation	cGAS knockout promotes ferroptosis.	^89^
	siRNA/CRISPR–Cas9	cGAS	HeLa cells transplanted into immunodeficient mice	cGAS–STING	Bax, SMAC, cytochrome *C*	cGAS knockout inhibits apoptosis.	^84^
STING agonists (DMXAA, ADU‐S100 and MSA‐2)	Cre–loxP system	STING1	Pancreatic ductal adenocarcinoma mice	NC‐STING	IRF3, IL‐35, IL‐10	NC‐STING pathway regulates the expression of Breg cells.	^86^
Construction of stable cell lines mediated by lentivirus	SPOPmut	STING1	Prostate cancer mice	NC‐STING–NF‐κB	STING1, IRF3, IFN‐β, NF‐κB	SPOP mutation up‐regulates NC‐STING–NF‐κB pathway.	^85^
PARPi		DNA	Triple‐negative breast cancer cell line	NC‐STING–NF‐κB	SSBs, DSBs, STING, IFN, NF‐κB	DNA damage promotes cGAS–STING pathway.	^12^
2‐BP	shRNA/CRISPR–Cas9	STING	RCC cell line	STING–VDAC2–mitochondrial calcium/ROS	ROS, mitochondrial calcium levels, ATP	Inhibiting or knocking out STING leads to the influx of calcium ions into mitochondria.	^10^
LNPs		cGAMP	Prostate cancer mice	STING pathway	CD8^+^ T cells, NK cells, IFNβ	cGAMP promotes STING pathway.	^40^
	Trp‐restriction	Trp	BCa cell line	Trp–IDO1–Kyn, Kyn–AhR–STING	STING, TBK1, IFN	Trp‐restriction promotes STING protein accumulation.	^107^
	CRISPR–Cas9	IDO1	BCa cell line	Trp–IDO1–Kyn, Kyn–AhR–STING	Kyn, STING, IFN	Inhibiting Trp catalysis promotes STING protein accumulation.	^107^
	CRISPR–Cas9	cGAS/STING	BCa cells obtained from bladder tumour samples	cGAS–STING–SLC14A1^+^ CAFs	GAS, STING, TBK1, IFN‐Is, SLC14A1, WNT5A	The knockout of cGAS or STING reduced the proportion of SLC14A1+ CAFs.	^74^

Abbreviations: BCa, blader cancer; CAF, cancer‐associated fibroblasts; CRISPR–Cas9, clustered regularly interspaced short palindromic repeats associated protein 9; DSBs, double‐strand breaks; DRP1, dynamin‐related protein 1; IDO1, indoleamine 2,3‐dioxygenase 1; Kyn, kynurenine; LNPs, lipid nanoparticles; ROS, reactive oxygen species; RCC, renal cell carcinoma; SSBs, single‐strand breaks; Trp, tryptophan.

## 10 CONCLUSION

This review delineates the context‐dependent duality of the cGAS–STING pathway in three primary urological cancers: PCa, RCC and BCa. The pathway regulates tumour progression through mechanisms involving inflammatory responses, cell death, TME, Trp metabolism and calcium homeostasis. Based on these functions, we summarise several urological tumour‐specific strategies targeting this pathway, including metabolic interventions (Trp deprivation/IDO1 inhibitors),^107^ delivery systems (RC@RMPs,^93^ LNPs^40^), palmitoylation inhibitors (2‐BP)^10^ and DNA damage repair inhibitors (PARPi).^101^ These approaches converge on cGAS–STING pathway, effectively alleviating CD8⁺ T cell exhaustion and mitigating immune evasion in multiple malignancies.

However, compared with other tumour systems, the cGAS–STING pathway exhibits unique context‐dependent properties in urological cancers. The high expression of AR and X chromosome‐linked genetic factors in urological malignancies interacts with the cGAS–STING pathway, offering novel therapeutic insights.^109^ Furthermore, BCa, characterised as an immunologically ‘cold’ tumour, shows direct regulation of cGAS–STING signalling by Trp metabolism.^107^ These features underline the distinctive crosstalk between urological cancers and the cGAS–STING pathway.

Our findings establish the first systematic comparison of tissue‐specific STING regulation in PCa/RCC/BCa, revealing its bidirectional role. Additionally, this context systematically reviews various avenues targeting cGAS–STING pathway in urologic tumours, whereas the clinical efficacy of these strategies remains to be verified. Of note, the long‐term safety of the cGAS–STING pathway remains an area of active investigation, requiring advances in single‐cell spatial biology, nanopharmacology and computational immuno‐oncology. This review provides a feasible strategy for enhancing the sensitivity of urologic tumours in clinical practice and extends the clinical benefits of immuno‐oncology approaches.

## AUTHOR CONTRIBUTIONS

**Q. Wei**: Conceptualisation, writing—original draft, methodology, editing and supervision. **K. Zhao**: Conceptualisation, editing and supervision. **Y. Wu**: Conceptualisation, editing and supervision. **W. Wu**: Conceptualisation and editing. **N. Hao**: Conceptualisation, editing, supervision, funding acquisition, methodology and validation.

## CONFLICT OF INTEREST STATEMENT

The authors declare conflicts of interest.

## ETHICS STATEMENT

Ethical approval is not applicable to this article as it is a review paper.

## Supporting information




Supporting information

## Data Availability

Data sharing is not applicable to this article as no datasets were generated or analysed during the current study.
