# Supplementary material for: Beyond tumour suppression: cGAS‐STING pathway in urologic malignancies: Context‐dependent duality and therapeutic implications
Source: Clin Transl Med. 2025 Nov 23;15(11):e70531. doi: 10.1002/ctm2.70531 (PMC12640615; doi:10.1002/ctm2.70531)
Supplement: Supplementary file 1 — Supporting information [file CTM2-15-e70531-s001.docx]

This narrative review synthesizes emerging insights into the bidirectional regulation of the cGAS-STING pathway in urologic malignancies to provide a translational framework for precision oncology. The methodology was as follows:

**Databases:** PubMed, Web of Science, Scopus.

**Search Period:** Up to August 2025.

**Keywords:** Combinations of "cGAS," "STING," "cGAS-STING," "prostate cancer," "bladder cancer," "renal cell carcinoma," "urologic malignancies," "immunotherapy," and "sex hormones."

**Selection Process:** Following a PRISMA-inspired screening process, our initial search yielded approximately **5000** articles. After removing duplicates, **1500** records underwent title and abstract screening, followed by a full-text review of **800** articles. **300** key studies were ultimately included.

**Synthesis:** The findings from the included studies were narratively synthesized to map the current evidence.
